# Fabry disease in females: organ involvement and clinical outcomes compared with the general population

**DOI:** 10.1186/s13023-025-03922-x

**Published:** 2025-08-13

**Authors:** Robert J. Hopkin, Dawn Laney, Sean Kazemi, Angela Walter

**Affiliations:** 1 Cincinnati Children’s Hospital Medical Center Division of Human Genetics, Department of Pediatrics, University of Cincinnati College of Medicine, 3333 Burnet Avenue, Cincinnati, OH 45229-3026 USA; 2 Division of Medical Genetics, Department of Human Genetics, Emory University School of Medicine, 101 Woodruff Circle, Atlanta, GA USA; 3 US Medical Affairs, Rare Diseases, Sanofi, Cambridge, MA USA

**Keywords:** Fabry disease, Lysosomal storage disorders, Females, Organ involvement, General population

## Abstract

Fabry disease (FD) is a rare, X-linked, progressive multi-system disorder of glycosphingolipid metabolism that causes cellular and organ damage in multiple body systems. FD has not been studied as extensively in females as in males due to greater heterogeneity of presentation and variability of disease course in females. Furthermore, despite published evidence to the contrary, females are still often referred to as carriers of FD and their symptoms assumed to be mild. Findings from recent studies and patient registries show that over two-thirds of females with FD experience signs and symptoms in different body systems, with over a third experiencing severe clinical manifestations. Symptoms include a wide variety of cardiovascular, neurologic, kidney, gastrointestinal, and psychiatric/psychologic effects, which significantly impair health-related quality of life and shorten life expectancy in affected females. Accurate and timely diagnosis is hindered by overlap of signs and symptoms (which may be non-specific) with other conditions, as well as lack of physician awareness. Females with FD are often compared with their affected male counterparts as opposed to unaffected females in the general population, which may result in less rigorous management for females than may be appropriate were they not being contrasted with males. It is more clinically appropriate to consider onset and severity of symptoms in females with FD in comparison to their unaffected counterparts in the general population. There is, therefore, a need for greater representation of females in clinical studies that are designed and powered to specifically detect endpoints in this group, and to evaluate these endpoints against those seen in females in the general population without FD. Improvements in the understanding of disease phenotypes, biomarkers, presentation, course, and outcomes in pediatric and adult females are needed.

## Background

Fabry disease (FD) is a rare, inherited (X-linked), progressive multi-system disorder of glycosphingolipid metabolism. Deficiency or absence of α-galactosidase A (α-Gal A) activity due to pathogenic variants in the *GLA* gene results in the lysosomal accumulation of globotriaosylceramide (GL3) and its deacylated derivative (globotriaosylsphingosine [lyso-GL3]) in a variety of cell types [1, 2]. The resulting cell and organ damage can manifest in multiple body systems [1].

**Fig. 1:**
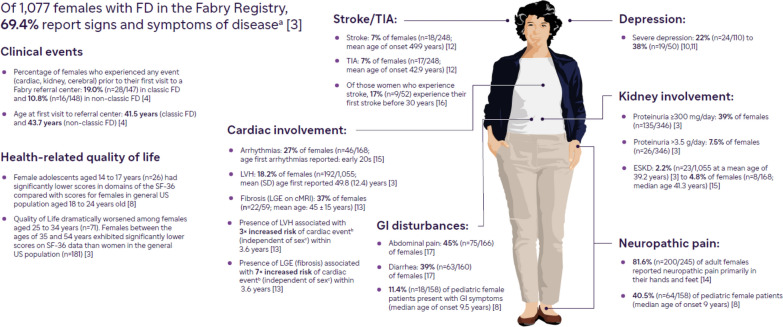
Summary of symptoms and organ involvement characteristics in female patients with classic FD [3, 4, 8, 10, 12–17]. cMRI; cardiac magnetic resonance imaging; ESKD, end stage kidney disease; FD, Fabry disease; GI, gastrointestinal; LGE, late gadolinium enhancement; LVH, left ventricular hypertrophy; MRI, magnetic resonance imaging; SF-36, Short Form 36 Health Survey Questionnaire; TIA, transient ischemic attack. ^a^Data cited above was pulled from multiple registries as well as academic studies. ^b^Cardiac events are defined as ventricular tachycardia, bradycardia requiring device implantation, severe heart failure, and cardiac death [52]. ^c^Sex did not modify the relationship between the composite end point (cardiac events) and any of the cardiac MRI parameters, including LVH (*P* = 0.15 for interaction term) and LGE (*P* = 0.38 for interaction term) [52]

Although FD affects both sexes, the disease has not been studied as extensively in females as in males due to the greater heterogeneity of presentation and variability in disease course in this group. Historically, females were thought to be only carriers of FD, and their symptoms, if present, were believed to be typically mild [2]. However, evidence from the literature demonstrates that females with FD often experience significant disease-related complications including cardiac involvement, cerebrovascular events, declining kidney function, gastrointestinal (GI) symptoms, neuropathic and abdominal pain, and reduced quality of life [3–5]. Moreover, reliance on patient recall in studies has led to underappreciation of the early age of symptom onset in females, with median age at diagnosis typically being many years later than the age of onset of symptoms [3]. In addition, asymptomatic females with FD may experience silent progression of injury to the kidneys, heart, or central nervous system (CNS) that without appropriate clinical assessments frequently goes undetected [6]. Furthermore, despite normal creatinine levels, eGFR > 60 ml/min/1.73m^2^ and minimal proteinuria, many female patients who have kidney biopsy studies show significant histologic evidence of FD [7]. Compared with the general population, females with FD have poorer quality of life and reduced overall survival [3, 6, 8, 9]. Figure 1 summarizes symptoms and organ involvement reported by female patients with FD [3, 4, 8, 10–17].

Much of the current literature on FD compares outcomes in mixed populations of male and female patients. The purpose of this review is to highlight the experiences of females living with FD when compared with age-matched females without FD in the general population. Organ involvement in females with FD are discussed, as well as, patient-reported outcomes and an overview of patterns of morbidity relative to the general population.

## Challenges in diagnosing and characterizing FD in females

Diagnosis and classification of FD are based on various factors including disease phenotype, α-Gal A activity, *GLA* genotype, and other biomarkers. This process is more challenging in females than in males because of heterogeneity in residual levels of α-Gal A, biomarkers, and disease presentation in this group [6, 18]. Disease expression in females depends on a variety of factors that include pathogenic variant, pattern and distribution of X-inactivation, clonal expansion, and functional mosaicism, making the condition arguably different in females than males. The pattern of inheritance does not fit well with either X-linked dominant or recessive [19]; therefore, it is more appropriate and desirable to refer to it as simply X-linked. Patterns of X-inactivation combined with absent or inefficient cross-correction between Fabry and non-Fabry cells in heterozygotes contribute to poor correlation between clinical manifestations of FD and plasma levels of α-Gal A in females [18, 20].

The wide range of clinical manifestations of FD may overlap with chronic and often more generally recognized conditions such as diabetes, multiple sclerosis, rheumatoid arthritis, bowel diseases (e.g., Crohn’s disease, irritable bowel syndrome, ulcerative colitis) and other lysosomal storage disorders. Consequently, if there is no family history of FD, correct diagnosis can be delayed by several years due to the non-specific nature of symptoms and lack of physician awareness [21, 22]. The Fabry Outcome Survey, one of several global, Fabry-specific rare disease registries, reported a mean delay from onset of symptoms to correct diagnosis of 16.3 years in 61 females with FD [21].

Accurate and timely diagnosis allows for initiation of appropriate and supportive management, genetic counseling and the possibility of informed reproductive choices, and effective treatment when necessary. It enables the possibility of identifying at-risk family members before the onset of irreversible organ damage. The importance of timely diagnosis is underlined by recent prospective and retrospective studies in very young children with FD that suggest symptoms such as neuropathic pain and GI issues in females may begin well before 5 years of age [23, 24]. Fabry Registry data from 352 children aged from infancy to 17 years documented a median age of 9 years for the onset of symptoms (notably pain, GI symptoms and impaired quality of life) in females and a median age at diagnosis in females of 9 years [8]. The relatively young age at diagnosis reflects the fact that most patients had family members previously diagnosed with FD [8].

### The value of rare disease registries

- As with any rare disease population, patients with FD are geographically dispersed. Subsequently, the experience of individual healthcare professionals caring for small numbers of patients with FD may not be sufficient to adequately characterize all aspects of the disease.
- Disease-specific registries, including but possibly not limited to the Fabry Registry, the Fabry Outcome Survey, FollowME Fabry Registry and the Italian Fabry Disease Cardiovascular Registry (IFDCR) have the benefit of following large numbers of patients at centers worldwide. These registries are essential in defining the long-term natural history of FD by allowing assessments of large groups of patients over time and across a spectrum of clinical phenotypes.
- The Fabry Registry (NCT00196742) is an on-going, international, multi-center observational program that collects retrospective and prospective patient data with the primary objectives of understanding disease variability and progression in the natural history period and characterizing long-term treatment outcomes.
- Participation is voluntary and open to patients with all types of FD, regardless of symptoms and whether or not they are receiving disease-specific therapy.
- The Fabry Registry contains data from more than 8000 individuals with Fabry disease from all over the world [25]. It currently enrolls patients from 245 centers across 45 countries/regions globally.
- Oversight of the Fabry Registry is the responsibility of international and regional Boards of Advisors comprised of independent physicians with extensive scientific and clinical FD expertise. These Boards ensure the highest standards of epidemiological and biostatistical methods are followed during the analysis and publication of data. Fabry Registry data are observational in nature and submitted voluntarily.
- It must be acknowledged that not all females with FD have been diagnosed and subsequently enrolled. However, the Fabry Registry team uses specific modeling analyses in an effort to account for this limitation [25].
- The Fabry Outcome Survey (FOS) was a worldwide survey completed in 2021. Patients enrolled in the FOS, were recruited from 144 centers across 26 countries.

Diagnosis of FD in females is also hindered by a lack of awareness of symptoms. In a Spanish cross-sectional study of 33 females with untreated FD (mean age 46 years), 22 females had at least one clinical symptom of FD, despite considering themselves to be asymptomatic [26]. Most frequent symptoms included tingling in hands and feet (41%); palpitations, constipation, joint pain, headache, and dizziness (27% for all); and hypohidrosis (23%) [26]. A recent study of 41 women with FD in Romania reported the presence of acroparesthesia in 55% of the women, but prior to their FD diagnosis, their pain was often interpreted as rheumatic and therefore not recognized as a symptom of Fabry disease until the emergence of more prominent clinical events [27]. The degree of organ involvement can also differ among women with the same underlying *GLA* variant and within the same family [28, 29], which may lead to a perception that they are asymptomatic when they actually have disease manifestations that differ from those of other family members. It is therefore important that females with FD have ongoing, multi-system monitoring, regardless of genotype, initial presentation, historical medical needs, or lack of events in other female relatives.

### FD phenotypes in females

Fabry disease in females can be divided into classic and non-classic phenotypes [4, 6]. In a multicenter study of 295 females, 147 had a classic phenotype defined as *GLA* variant and one or more FD-specific symptoms. FD-specific symptoms were defined as Fabry neuropathic pain, angiokeratoma and/or cornea verticillate [4]. The other 148 females had a non-classic phenotype defined as *GLA* variant without characteristic FD-specific symptoms. The presentation of these phenotypes varies according to several factors including genotype classification: in females with classic disease, symptom onset will typically be in childhood or adolescence, compared with the fourth to seventh decade of life in females with non-classic disease [6]. There is typically early onset multi-organ involvement in classic FD, whereas non-classic disease may have fewer symptoms and is often recognized because of predominant involvement of heart or kidney damage [6]. There is often evidence of multi-organ involvement in females with non-classic FD when detailed evaluation is performed [30].

Presenting symptoms differ between females with classic versus non-classic disease. Neuropathic pain, angiokeratomas, corneal/lenticular opacities, hypohidrosis and GI disturbances starting early in life, are characteristic of a classic phenotype. Females with non-classical FD may present much later and some will have predominantly cardiovascular symptoms [6, 31]; however, non-cardiac involvement is also frequently seen in non-classical cases [4, 32]. The rate of clinical events in females with the classic phenotype is generally higher than in females with non-classic disease [4]. The potential for crosstalk between heart and kidney in non-classic disease is unknown because of lack of long-term data.

### FD genotype–phenotype correlations

The accurate prediction of phenotype in females with FD cannot be based on genotype alone. Significant phenotypic heterogeneity exists in females with FD and therefore defining FD in females by genotype–phenotype correlations is complex. It has been hypothesized that X-inactivation in females is a key factor in determining phenotype, but recent studies exploring the correlation between X chromosome inactivation patterns and symptom severity have been inconsistent. X-inactivation is the process of randomly silencing one X-chromosome in females in order to ensure equivalent gene dosage between females with two X-chromosomes and males with one X-chromosome. Blood samples from females with FD frequently reflect skewed X chromosome inactivation patterns, but these patterns seen in plasma do not reliably correlate with disease severity, α-Gal A activity or lyso-GL3 levels [33]. Organ-specific, non-random X-chromosome inactivation in females who have inherited a *GLA* variant influences the levels of α-Gal A activity in that cell and may thus contribute to phenotypic variation [18]. Unlike males, assessment of α-Gal A activity in plasma in females is not predictive of disease burden as females with balanced X-inactivation and normal or near normal α-Gal A activity in plasma will nevertheless often present with disease manifestations [6, 18].

By comparison, in the case of Mucopolysaccharidosis II (MPS II), while also an X-linked lysosomal storage disorder whose deficit of lysosomal enzyme iduronate 2-sulphatase results in accumulation of glycosaminoglycans in multiple organ systems [34], female carriers of pathogenic variants in iduronate 2-sulfatase (*IDS*) are rarely affected unless there is a simultaneous presence of two mutant alleles or another coincidental genetic impact [35]. One explanation for this absence of symptoms in females with pathogenic *IDS* variants versus the presence of symptoms in the majority of females harboring pathogenic *GLA* variants is the mechanism of cross-correction. Cross-correction is the cellular process whereby functional enzyme secreted by cells in which the wild-type allele is active is taken up by neighboring cells through the mannose-6-phosphate receptor pathway, thereby correcting the metabolic deficiency [35, 36].

In 2015, Fuller and colleagues demonstrated successful cross-correction of the enzymatic defect in vitro in fibroblasts from patients with MPS II, resulting in the reduction of glycosaminoglycan storage to the levels detected in control cells. In contrast, their work revealed a minimal secretion of mature alpha-galactosidase A (GLA) rather than mannose 6-phosphylated GLA from unaffected FD fibroblasts into intracellular spaces or plasma. Subsequently, enzymatic cross-correction of affected cells in Fabry fibroblasts did not occur [36].

More recent morphometric studies in kidney biopsies from females with FD have shown podocyte^1^ cytoplasm containing GL-3 to be identical in male and female Fabry patients, suggesting that Fabry podocytes in females do not benefit from the enzymatic activity of adjacent non-Fabry podocytes or from higher residual plasma α-Gal-A; thus, dispelling the hypothesis that females with Fabry disease might benefit from enzymatic cross-correction [38]. Taken together, these findings further support the lack of evidence of enzyme cross-correction between cells expressing α-Gal A and affected cells without α-Gal A activity in heterozygous females [6, 39]

Over 1000 *GLA* variants contribute to the extensive heterogeneity of FD [40]. These include pathogenic variants associated with the classic phenotype or with non-classic disease, variants of unknown significance, and benign polymorphisms [6]. In addition, genes that modify the phenotype must also be considered. The large number of private variants makes the collection of sufficient data across enough patients for full phenotypic characterization challenging [41].The lack of cross correction between cells significantly complicates efforts to draw correlations between phenotype and plasma or leukocytic α-Gal A activity [12, 42]. No consistent correlation between phenotype and plasma or leukocytic α-Gal A activity has been identified from the limited data available [12, 30, 42].

In spite of the complexity and variability seen in females with FD, it is important to note that there is a clear differentiation in risk for major medical events between variants in the *GLA* gene associated with classical FD and those associated with non-classical FD [4].

### Biomarkers

Plasma levels of lyso-GL3, a biomarker used in diagnosis, can be useful for differentiating between classic and non-classic Fabry phenotypes. [43]. Lyso-GL3 levels are typically lower in females and while older research suggests lyso-GL3 levels may not correlate with genotypic severity in females as they do in males [4, 43, 44], newer studies with large cohorts of female patients have shown statistically significant associations between lyso-GL3 levels and risk of clinical events in females, including kidney replacement therapy, atrial fibrillation, pacemaker and/or implantable cardioverter defibrillator, cerebrovascular events or death [45].

Data published by van der Veen and colleagues in 2023 (*n* = 237; 62% females) has shown that plasma lyso-GL-3 levels in patients not treated with disease-specific therapy reach a stable level in childhood, remain unchanged over decades, and are associated with the severity and/or progression of nearly all measured Fabry disease manifestations in both females and males [46]. Measured Fabry disease manifestations included eGFR slope, progression of albuminuria, left ventricular (LV) mass, diastolic dysfunction, and progression of white matter lesions [46]. These observations highlight the importance of multi-organ assessment and ongoing monitoring in females with FD.

## Significant clinical events and life expectancy in females with FD

Of 1077 females enrolled in the Fabry Registry, 69.4% experienced signs and symptoms of FD [3]. Severe clinical manifestations have been reported by approximately 43% of females with FD [6]. Overall, proportions of females presenting with kidney (dialysis, kidney transplantation, two or more estimated glomerular filtration rate [eGFR] values < 60 mL/min/1.73 m^2^ ≥ 90 days apart or ≥ 50% increase in serum creatinine levels), cardiovascular (myocardial infarction [MI], significant cardiac procedures, arrhythmia, angina pectoris, congestive heart failure [CHF] or left ventricular hypertrophy [LVH] II), or cerebrovascular (transient ischemic attacks [TIA], hemorrhagic or ischemic stroke) signs of FD were 10.6%, 10.0% and 4.2%, respectively [3, 16]. Corresponding mean ages of onset were 30.9, 33.4, and 31.2 years [3]. Of a cohort of 341 untreated adult females in the Fabry Registry, 21% had experienced a major cardiac (MI, arrhythmia, cardiac syncope, CHF, angina pectoris, or significant cardiac procedure), cerebrovascular (stroke), or kidney event (long-term dialysis ≥ 40 days, kidney transplantation, or eGFR values < 10 mL/min/1.73 m^2^) [47].

Clinical events are not limited to females with classic FD but may also occur in females with non-classic FD as reported by Arends et al., 2017. (Table 1) [4]. Females with FD also experience a range of other manifestations that can have a significant negative impact on quality of life, including GI symptoms, angiokeratoma, hypohidrosis, acroparesthesia, lymphedema, severe chronic fatigue, and auditory and ocular symptoms [6].

**Table 1:** Characteristics of adult (≥ 18 years) female patients with classic and non-classic Fabry disease participating in a multicenter European study [4]

Characteristic	Classic phenotype *n* = 147	Non-classic phenotype *n* = 148
Mean age at first visit (SD, range)	41.5 (13.5, 18–75)	43.7 (15.0, 18–79)
Any event before first visit	28 (19.0%)	16 (10.8%)
Cardiac event before first visit	11 (7.5%)	9 (6.1%)
Arrhythmia-related	9 (6.1%)	5 (3.4%)
Ischemia-related	2 (1.4%)	4 (2.7%)
Kidney event before first visit	2 (1.4%)	1 (0.7%)
Kidney transplant	1 (0.7%)	1 (0.7%)
eGFR < 15 mL/min/1.73 m^2^	1 (0.7%)	0
Cerebral events before first visit	16 (10.9%)	6 (4.1%)
TIA	9 (6.1%)	5 (3.4%)
Stroke (symptoms > 24 h)	7 (4.8%)	1 (0.7%)
eGFR < 60 mL/min/1.73 m^2^ before first visit	8/142 (5.6%)	14/147 (9.5%)
Left ventricular hypertrophy	59/132 (44.7%)	40/128 (31.2%)
Concentric hypertrophy	47/132 (35.6%)	32/128 (25.0%)
Concentric remodeling	18/132 (13.6%)	17/128 (13.3%)
Median lyso-GL3, nmol/L (range)	9.3 (0.7–4.2)	2.5 (0.4–20)

Table taken from Arends M, et al. Characterization of Classical and Nonclassical Fabry Disease: A Multicenter Study. *JASN.* 2017;28(5):1631–1641 [4]

eGFR, estimated glomerular filtration rate; FD, Fabry disease; lyso-GL3, globotriaosylsphingosine; SD, standard deviation; TIA, transient ischemic attack

### Life expectancy in FD

Life expectancy for females with FD has been reported as 75.4 years in a cohort of 1,426 females in the Fabry Registry [9], while in another cohort of 754 females in the Fabry Outcome Survey, mean age at death was 64.4 years (Table 2) [21]. This represents a reduction of 3.7–14.7 years relative to the US general population (Table 2).

**Table 2:** Life expectancy in females with FD versus females in the general population [9,21, 48]

Outcome	Females with FD	Females in the general population
Life expectancy	75.4 years (*n* = 1426; Fabry Registry) [9] 64.4 years (*n* = 754; Fabry Outcome Survey) [21]	79.1 years (NVSS, Mortality data 2022) [48]
Years of life lost	3.7 years^a^ (*n* = 1426; Fabry Registry) [9, 48] 14.7 years^b^ (*n* = 754; Fabry Outcome Survey) [21, 48]	N/A

FD, Fabry disease; NVSS, National Vital Statistics System

^a^Loss of life expectancy calculated as 79.1–75.4 years. ^b^Loss of life expectancy calculated as 79.1–64.4 years

### Cardiac manifestations in females with FD

The pathogenic mechanisms underlying cardiac damage in FD are diverse and not fully characterized, but can include conduction abnormalities, arrhythmias, LVH, ischemia, cardiomyopathy, diastolic dysfunction, valvular dysfunction, and heart failure [6, 49–51]. The earliest cardiac finding related to Fabry disease is bradycardia which can be seen in childhood in both females and males [52]. Early asymptomatic cardiac involvement in adulthood may be signaled by LVH, diastolic dysfunction, and conduction abnormalities (PQ/PR interval changes and resting bradycardia), while mid-stage symptoms include myocardial fibrosis, microvascular angina, and valvular heart disease [6, 49, 53, 54]. Patients with late-stage cardiac involvement can experience reduced exercise tolerance, arrhythmias, heart failure, and sudden cardiac death, typically during the fourth or fifth decade of life [6, 55]. The risk of cardiac involvement and the importance of screening for FD among females with cardiomyopathy were highlighted by the diagnosis of non-classic FD in four (12%) of 34 women with hypertrophic cardiomyopathy (mean age: 51.5 years) with no family history of FD, who participated in a study in which tissue samples were analyzed for histology and by electron microscopy [20]. These findings underscore the importance of considering Fabry disease in women with otherwise unexplained LVH.

Cardiac involvement in females with classic FD is often asymptomatic and may be advanced and less responsive to management by the time it manifests clinically [54]. The need for concern in females with non-classic disease was shown by results in 126 women with a mean age of 41 years with the non-classical N215S (p.Asn215Ser missense) *GLA* variant, who were retrospectively compared with 237 females with classic variants [56]. Cardiac parameters (interventricular septum thickness and LV posterior wall thickness [LVPWT]) were generally similar in females with classic and non-classic variants, but older females (65–74 years) with classic disease were more likely to have abnormal LVPWT values (> 10 mm) than those with the N215S variant [56]. Cardiac events (angina pectoris, arrhythmia, CHF, MI, implantation of cardiac devices, or significant cardiac procedures) were experienced by 8% and 15% of females with N215S or classic genotypes, respectively [56].

Eleven Taiwanese females with a well characterized IVS4 + 919G > A splice-site variant were compared with 12 women with classic variants [57]. Frequencies of LVH (91 vs. 92%, respectively) and arrhythmias (55 vs. 58%) were similar in both groups, but with later mean ages of onset for patients with the non-classical IVS4 variant (LVH: 57 vs. 50 years; arrhythmia: 63 vs. 49 years) [57]. The authors of the Asian Fabry Cardiomyopathy High-Risk Screening Study (ASIAN-FAME) suggested that late-onset IVS4 + 919G > A FD should be considered in reviewing root causes of LVH [58].

### Common cardiovascular manifestations and events

Cardiac involvement is the most common serious manifestation of FD in females, reported in 59% (147/248) of women at a mean age of 33.5 years ± 18.1 years [12]. A retrospective Spanish study of 97 females with FD (mean age 50.1 years; classic [40.2%], non-classic [53.6%]) showed that cardiac involvement was the most prevalent major organ involvement in 49.5% of females, and the primary reason for initiation of FD-specific treatment [59]. Of the 34 untreated females included in the study who had major organ involvement (56.7% of total untreated), 27 had cardiac, kidney, or cerebrovascular manifestations [59]. In a retrospective chart review of 168 females with predominantly classic FD spanning a median of 12 years per patient, 35% (59/168) of females with FD experienced a cardiac event or required a cardiac intervention by a mean age of 44.4 years. Events and interventions included MI, cardiac procedures, angina, heart failure and arrhythmias [15]. A more recent study of 98 females with classic FD, median age 50 years, reported major cardiovascular events (MACE: including cardiovascular death, hospitalization for heart failure, sustained ventricular arrhythmia, or MI) were experienced by 14% (14/98) females [60].

Cardiovascular events, including atrial fibrillation (AF), which increases the risk of stroke if not managed adequately [60, 61], occur more frequently and earlier in females with FD than in the general US population (see Table 3 for details), and incidence increases with age.

**Table 3:** Cardiac manifestations in females with FD versus females in the general population [3, 9, 12, 13, 15, 52, 60, 64, 65, 67, 79–88]

Outcome	Females with FD	Females in the general population
CV death	• 57% (*n* = 4/7; Fabry Outcome Survey; median age: 64.4 years) [80] • 50% (*n* = 5/10; Fabry Registry; median age: 66.0 years) [9]	21.8% (all ages) [82] 16.3% (45–64 years) [82] 19.9% (64–84 years) [82] 21.8% (*n* = 6,203/28,501) [81]
Cardiac involvement and/or CV disease	• 59% (defined as chest pain, palpitations, LVH; *n* = 147/248; Fabry Outcome Survey; median age at onset: 33.5 years) [12]	44.8% (defined as coronary heart disease, heart failure, stroke, and hypertension; ≥ 20 years old; US) [83] 9.2% (defined as coronary heart disease, heart failure, and stroke [excluding hypertension]; ≥ 20 years old; US) [83] 10.1% (defined as heart disease [excluding coronary heart disease-specific, hypertension, and stroke]; ≥ 20 years old; US) [83]
MI	10%^a^ (*n* = 10/98; median age at first event: 51 years; Netherlands) [60] 1.7% (*n* = 18/1055; Fabry Registry; median age at first event 56.2 years) [3] 1.6–4.3% (untreated *n* = 4/254; treated n = 5/115; Fabry Outcome Survey; mean age of event: untreated, 59.3; treated, 58.1 years) [65] 2% (*n* = 3/168; mean age: 44.9 years; US, Canada, Europe) [15]	0.7–2.3% (≥ 20 years old); 0.7% non-Hispanic Asian; 2.0% non-Hispanic White; 2.1% Hispanic; 2.3% non-Hispanic Black; [84, 85] Mean age at first MI: 72 years [86]
Arrhythmia	27% (*n* = 46/168; mean age: 44.9 years; US, Canada, Europe) [15] 21% (*n* = 3/14; Fabry Registry; aged < 18 years) [52] 7.1% (*n* = 75/1055; Fabry Registry; median age at first arrhythmia: 47.0 years) [3]	0.12% in pediatric population < 18 years (median age: 10.4 years)^b^ [87] 1.6% (*n* = 1840; 55–64 years; UK) [88] 3.2% (*n* = 1540; ≥ 65 years; UK) [88]
Atrial fibrillation	16%^a^ (*n* = 16/98; median age at first event: 58 years; Netherlands) [60] 3%^c^ (*n* = 1/32; median age at first event: 68 years; Netherlands) [60]	0.1% (*n* = 530; < 55 years) [64] 0.4% (*n* = 310; 55–59 years) [64] 9.1% (*n* = 1132; ≥ 85 years) [64] 0.9% (*n* = 1035; 55–64 years; UK) [88] 2.1% (*n* = 1027; ≥ 65 years; UK) [88]
Heart failure	6% (*n* = 6/98; median age at first event: 69 years)^a^ (Netherlands) [60] 2.9% (*n* = 31/1055; Fabry Registry; median age at first report: 54.0 years) [3] 1.2% (*n* = 2/168; mean age: 44.9 years; US, Canada, Europe) [15]	0.7–3.3% (≥ 20 years old) [84, 85]
Myocardial fibrosis	LGE on cMRI: 37% (*n* = 22/59; mean age: 45 years ± 15; Canada) [13]	No female general population data available
LVH	26% (*n* = 65/248; Fabry Outcome Survey; median age at onset: 50.4 years) [12] 18.2% (*n* = 192/1077; Fabry Registry; median age first reported: 51.0 years) [3] 21.3–37.4% (untreated *n* = 54/254; treated *n* = 43/115; mean age at event: untreated, 48.8; treated, 50.1 years) [65] 24% (*n* = 8/34; median age: 48 years) [79]	9.1% (*n* = 1420; mean age: 54.6 years; Norway) [67]

cMRI, cardiac MRI; CV, cardiovascular; FD, Fabry disease; LGE, late gadolinium enhancement; LVH, left ventricular hypertrophy; MRI, magnetic resonance imaging

^a^In females with classic FD. ^b^Based on a mixed population of females and males. ^c^In females with non-classic FD

#### Arrhythmias

Arrhythmias, most commonly sinus bradycardia, are among the first cardiac manifestations reported in FD, and may begin in childhood, with 21% of female pediatric patients being affected (*n* = 2/14; Fabry Registry data; Table 3) [52]. Arrhythmias are also the most common cardiac event in adult females with FD, with onset in the third decade, and approximately half are symptomatic [15]. Bradyarrhythmias can cause syncope, light-headedness/dizziness and near syncope, CHF, exercise intolerance, fatigue, and confusional states [62], and FD is known to be associated with significant impairment (linked to bradycardia and diastolic dysfunction) of exercise tolerance [63]. The prevalence of arrhythmias in the FD population should be considered when prescribing drugs for other FD symptoms.

In a cohort of females with FD (98 classic, 32 non-classic) in the Netherlands, 16% with classic and 3% with non-classic phenotype had AF, with median ages of onset 58 and 68 years, respectively [60]. For comparison, the overall prevalence of AF in the female general population of the US has been estimated at 0.8% overall, being highest (9.1%) in women aged ≥ 85 years (see Table 3) [64].

#### LVH and fibrosis

LVH manifests in 18.2% to 37.4% of females with FD at a median age of approximately 50 years, with severity correlating strongly with age [3, 65, 66]. In contrast, data from 1420 females suggest LVH in only 9.1% in the Norwegian general population, with a similar age of onset (mean 55 years; Table 3) [67].

LVH is associated with increased risk of cardiac events such as arrhythmia, heart failure and death [13], and the degree of LVH and presence of myocardial fibrosis are risk factors for malignant ventricular tachyarrhythmias and sudden cardiac death [68]. Ventricular changes should be assessed individually over time and diagnostic parameters should be used against the background of other comorbidities in females. For example, routine screening of *GLA* sequencing has been recommended in females with unexplained LVH older than 40 years and with unknown family background [69].

In women with Fabry disease, loss of myocardial function and development of fibrosis does not require the presence of LVH. In a study of 58 women with FD (mean age, 42 years), when LV mass was examined using cardiac magnetic resonance imaging (cMRI) the presence of late gadolinium enhancement (LGE) indicating fibrosis was detected in 23% of the female patients without hypertrophy. In this study, the youngest woman to develop fibrosis was 36 years old while LVH was not observed in patients aged younger than 46 years [70]. In another cMRI study of 125 females with FD, the presence of LGE was not associated with LVH (age-adjusted OR, 1.26; 95% CI 0.44–3.53; *p* = 0.66) [4]. Data from a Taiwanese program screening for the late-onset IVS4 + 919G > A mutation showed significant cardiomyocyte substrate accumulation leading to severe and irreversible cardiac fibrosis before the development of LVH and other morbidities [71]. Imaging showed significant LGE in the absence of LVH in 16.7% of IVS4 + 919G > A-positive women, highlighting the danger of insidious, ongoing, and irreversible cardiac damage in patients with late-onset FD [71].

In a cohort of female patients with FD (mean age 45 years) who had undergone cMRI at a single large tertiary referral hospital in Canada between March 2008 and January 2019, fibrosis (LGE on cMRI) was present in 37% (*n* = 22/59) of females [13]. In this study, it was shown that persons with FD and myocardial fibrosis have significantly worse cardiac-related event-free survival than those without fibrosis, regardless of sex [13], and the presence of fibrosis as indicated by LGE in cMRI independently predicts risk of cardiac events (adjusted hazard ratio 7.2; 95% CI, 1.5, 34; *p* = 0.01) versus absence of LGE [13]. Myocardial fibrosis has also been shown to increase the risk of ventricular arrhythmias in other conditions, such as hypertrophic cardiomyopathy [72]. In a cohort of 90 patients of mean age 44 years with gene-positive FD who underwent cMRI, LVH was also shown to be a strong predictor of adverse cardiac events, with both females (*n* = 59) and males (*n* = 31) with LVH having a threefold increase in risk, independent of sex, over a 3.6-year follow-up period [13]. The risk increased with left ventricular mass, with each 5 g/m^2^ increase associated with an 8% increase in event risk [13]. A validated risk prediction model based on cMRI parameters (including left ventricular mass) has been developed for use in routine clinical care to estimate 5-year risk of adverse cardiac outcomes in males and females with FD [73]. More recently, in a study of 268 patients with FD (53% female, age 50.4 ± 15.4 years), patients with severe LVH and impaired LV global longitudinal strain had the highest incidence for MACE (log-rank P < 0.05), irrespective of sex. The authors stressed the importance of sex-specific grading of LVH for most appropriate risk stratification in patients with FD [74].

Levels of cardiac troponin and *N*-terminal brain natriuretic propeptide (NT-proBNP) markers for myocardial damage and cardiac involvement may also be elevated in females with FD [75, 76]. High troponin and high NT-proBNP were reported in 25.9% (*n* = 28/108) and 37.3% (*n* = 38/102) of females with FD (mean age 44.1 years) [77].

Deleterious structural and functional myocardial changes in FD result in impaired cardiovascular function, which may contribute to exercise intolerance and fatigue [78]. In one study, exercise intolerance was experienced by 33 of 40 females (83%) of mean age 46.1 years with FD [79].

#### Cardiovascular mortality

Cardiovascular disease is the most common cause of death in females with FD [9, 80]. Cardiovascular death has been reported at rates of 50–57% at a median age of 64–66 years in females with FD compared with 21.8% in females in the US general population (all ages; Table 3) [7, 80, 81]. Broken down by age range, cardiovascular death is the cause of death in the general US population for 3.4% of females ages 1–19 years, 9.0% of females ages 20–44 years, 16.3% of females 45–64 years, 19.9% of females ages 64–84, and 27.7% of females 85 years and older [82].

### Stroke in females with FD

Neurologic symptoms of FD can occur throughout a patient’s life and affect both the peripheral nervous and CNS [1, 6, 89–91]. In the CNS, common cerebrovascular manifestations include TIA and stroke [12]. Moreover, there is a high prevalence of FD among female stroke patients. In a systematic review and meta-analysis (*n* = 8148), the prevalence of FD was 1.4% in female stroke patients of all etiologies, with higher rates observed in females with cryptogenic stroke (3.4%) [92].

Cerebrovascular events are more prevalent in FD than the general population (Table 4), with strokes reported in 7–22% of females with FD compared with 2.6% of the US general adult population, and TIA in 4–24% versus 1.4% in persons aged ≥ 55 years in the Netherlands [12, 15, 79, 93]. First stroke events in females with FD are also seen on average at much younger ages (43.1 mean age at first TIA (SD 15.5); 44.9 mean age at first stroke (SD 14.0) than in the US general population (81 years; Table 4) [3, 12, 15, 16, 79]. Of those women who experience stroke, 17% (*n* = 9/52) experience their first stroke before 30 years (median age 24.1 years) [16]. Early-onset strokes in females with FD are associated with increased risk of subsequent cardiac and kidney events; however, 76.9% of those with strokes experience a stroke before any cardiac or kidney events; 50% experience a stroke as their only clinical event; and 38.3% have first stroke before a diagnosis of FD [16].

**Table 4:** Stroke in females with FD versus females in the general population [3, 12, 15, 16, 79, 93, 95]

Outcome	Females with FD	Females in the general population
Stroke	7% (*n* = 18/248; Fabry Outcome Survey; mean age at onset: 49.9 years) [12] 22% (*n* = 8/36; median age: 48 years) [79] 8% (*n* = 14/168; mean age at first event: 44.9 years; US, Canada, Europe) [15] 4.3% (*n* = 52/1203; Fabry Registry; median age at first stroke: 45.7 years) [16]	2.6% (aged ≥ 18 years) [95]
TIA	24% (*n* = 9/37; median age: 48 years) [79] 7% (*n* = 17/248; Fabry Outcome Survey; mean age at onset: 42.9 years) [12] 3.6% (*n* = 6/168; mean age at first event: 43.1 years; US, Canada, Europe) [15] 3.9% (*n* = 41/1055; Fabry Registry) [3]	1.4% (≥ 55 years; Netherlands) [93]

FD, Fabry disease; TIA, transient ischemic attack

The presence of white matter lesions was shown to progress in 23.1% (*n* = 15/65) of female patients with FD over 46 months of follow-up (mean baseline age 42.1 years), with no effect of sex on prevalence or severity [94].

Atrial fibrillation (AF) has been shown to increase the risk of stroke as much as five-fold in the general population [61]. While not the primary driver of stroke in this population, the role of AF in the risk of stroke in FD should therefore also be considered, as AF is more common in females with FD than in the general population (Table 4) [60, 64].

### Kidney manifestations in females with FD

Females with FD may have podocyte and vascular damage before signs and symptoms of nephropathy become evident. Significant histologic changes (e.g., interstitial fibrosis and glomerulosclerosis) can occur before declining kidney function is detected via standard laboratory values [96]. In a study of 24 females with FD and clinically mild nephropathy, glomerulosclerosis and interstitial fibrosis were observed in patients with minimal or no proteinuria [96].

Females with FD show mosaicism in the kidneys with GL3 storage and non-storage podocytes. In a study of 12 female FD patients of median age 15 years, approximately 50% of podocytes per glomerulus were classified as having the storage phenotype, and the remaining 50% were non-storage (no GL3). There was a direct relationship between non-storage podocyte phenotype per glomerulus and age in females, consistent with little or no cross-correction between FD podocytes and non-FD podocytes [38]. When studied within age-matched pairs, females with lower proportions of non-FD podocytes had greater podocyte foot process width (an index of effacement) than those with higher proportions of non-FD podocytes [38]. No difference has been shown between age-matched males and females in terms of GL3 volume in FD podocytes, and accumulation of GL3 in these podocytes progresses with age in association with podocyte loss and proteinuria in females [39].

Albuminuria is one of the earliest clinical signs of Fabry-related kidney involvement and may precede manifest proteinuria and progressive kidney disease by many years. Albuminuria in females with FD should be considered a marker of potentially progressive vascular damage, warranting consideration of kidney biopsy in order to assess the extent of FD-related kidney damage present [97].

### Kidney function and ESKD in females with FD

Natural history data provides insights into the rates of kidney disease progression in females with FD. Of particular importance is the rate at which the slope of eGFR decline steepens in females with FD whose baseline eGFR is < 60 ml/min/1.73m^2^/year. In a retrospective analysis of 638 females with FD, 121 (19%) had an eGFR < 60 ml/min/1.73m^2^/year, among which 14.6 and 4.4% had chronic kidney disease stages 3 and 4–5, respectively (mean age: 51.8 years) [3]. In females with FD, eGFR declines significantly with increasing age [3].

Schiffmann and colleagues performed a comprehensive, retrospective chart review of 168 untreated females with FD [15], mean age 44.9 years (10.3–77.1), and found those females who developed end stage kidney disease (ESKD) (*n* = 4) had a mean progressive eGFR rate of -3.05 ml/min/1.73m^2^/year whereas females who did not develop ESKD (*n* = 51) reported a mean progressive eGFR rate of -1.02 ml/min/1.73m^2^/year. When stratified by chronic kidney disease status (CKD), progression rates were 2X greater for females who had baseline eGFR < 60 ml/min/1.73m^2^/year compared with those who had higher baseline eGFR values. Progression rates for females with baseline eGFR ≥ 60 ml/min/1.73m^2^/year were 0.9 ml/min/1.73m^2^/year, which is within the range expected for normal population eGFR decline over time, while progression rates for females with baseline eGFR < 60 ml/min/1.73m^2^/year were twice that slope at a rate of -2.1 ml/min/1.73m^2^/year. The mean age of females in this cohort with baseline eGFR < 60 ml/min/1.73m^2^/year was 52.1 years (SD 13.6).

Many females with FD experience decreased kidney function, exhibiting substantial levels of proteinuria. Proteinuria ≥ 300 mg/day over a 24 h period has been reported in 35%-39% of females with FD (*n* = 248, *n* = 346 respectively) [3, 11]. Baseline proteinuria is a critical predictor of kidney health, associated with a more rapid deterioration of kidney function when elevated. In the same previously referenced natural history study of 168 females with FD [15], females with baseline urine protein 0.1 g/24 h had a rate of eGFR slope of -0.66 ml/min/1.73m^2^/year. Females with FD whose baseline urine protein was between 0.1-1 g/24 h had a rate of eGFR progression of -2.2mk/min/1.73m^2^/year. In the same analysis, all eight FD females with overt baseline proteinuria (> 0.3 g/day) progressed to ESKD at a median age of 42.4 years [15].

In contrast, proteinuria is less common among females in the general population, as well as other higher-risk populations including children/adolescents with chronic kidney disease, and people with type 2 diabetes, although similar rates are seen in persons with type 1 diabetes [98, 99]. Proteinuria has been reported as a long-term outcome in 12.7% of living persons with a single kidney (although data specific to females were not available) [100].

Elevated serum creatinine levels, a late marker of kidney dysfunction, have been observed in females with FD, with a study in 40 females reporting elevated serum creatinine (> 1.2 mg/dL) in 15% of cases [101]. However, by the time serum creatinine is elevated, histologic damage is well underway. In a study of 13 female patients aged from 17–48 years with FD, four (31%) had greater than 50% foot-process effacement, a sign of podocyte injury despite normal creatinine values and minimal proteinuria [7]. This suggests that histopathologic assessment should be considered in females with normal baseline and serology levels to assess tissue involvement and assist with management decisions [7].

Females with FD are also at risk of developing premature kidney failure. Nephropathy progression and development of ESKD (which leads to a need for dialysis or transplant, or premature death) are more rapid in females with FD who present with decreased eGFR and proteinuria [3, 15]. In a study of females with classic (*n* = 147) and non-classic (*n* = 148) FD, the likelihood of a kidney event, the rate of progression of kidney disease based on eGFR, and the risk of developing ESKD were reported to be similar in both phenotypes [4].

Data suggest that ESKD requiring dialysis or transplantation occurs in 2.0–4.8% of females with FD at a mean age of 39–42 years [3, 15, 102], compared with a rate of only 0.2% for females in the general population (Table 5) [103]. Median time from first kidney replacement therapy to death for females with FD is 6 years [102] compared with 11.7 years for dialysis and 32.4 years for transplant in general population females with CKD [104].

**Table 5:** Kidney manifestations in females with FD versus females in the general population [3, 8, 12, 15, 60, 102, 103, 105–108]

Outcome	Females with FD	Females in the general population
Proteinuria	≥ 300 mg/day: 39% (*n* = 135/346; Fabry Registry; median age: 42.0 years) [3] Any level: 35% (*n* = 86/248; Fabry Outcome Survey; mean age at onset: 38.8 years) [12] > 150 mg/day: 11.4% (*n* = 5/44; Fabry Registry; aged 2–17 years) [8]	≥ 0.20 mg/mg (approximating 250 mg/day): 2.4% (≥ 25 years; Australia) [105] ACR ≥ 30 mg/g: 3.3%^a^ (*n* = 2845; aged 12–17 years) [106]
Chronic kidney disease	Stage 3: 14.6% (*n* = 93/638; Fabry Registry; mean age: 40.5 years) [3] Stage 4/5: 4.4% (*n* = 28/638; Fabry Registry; mean age: 40.5 years) [3] Stage 3: 13.4%^b^ (*n* = 11/82; median age at last visit: 51 years; Netherlands) [60] Stage 4/5: 1.2% (*n* = 1/82; median age at last visit: 51 years; Netherlands) [60]	Stage 1–4: 14.4% (≥ 18 years) [103] Stage 3–5: 5.8% (≥ 20 years) [107] Stage 3–5: 10.6% (≥ 18 years; UK) [108]
ESKD	4.8% (*n* = 8/168; median age at event: 41.3 years; US, Canada, Europe) [15] 2.0% (*n* = 27/1353; Fabry Registry; median age at first KRT: 38.0 years) [102] 2.2% (*n* = 23/1055; Fabry Registry; mean age at KRT: 39.2 years) [3]	0.2% (≥ 18 years)^c^ [103]

ACR, albumin–creatinine ratio; ESKD end-stage kidney disease; FD, Fabry disease; KRT, kidney replacement therapy

^a^Meta-analysis comprising 19 studies (44–66,616 participants). ^b^QUEST-RA project included 4,363 patients from 48 sites in 15 countries. ^c^Based on a mixed population of females and males

### Gastrointestinal symptoms, neuropathic pain, and reduced quality of life

Peripheral nervous system symptoms (chiefly neuropathic pain) and GI manifestations (along with skin complaints) are among the earliest signs and symptoms that occur in females with FD [8]. According to the Fabry Registry, neuropathic pain (including episodic pain crises, chronic pain and acroparesthesia) was a presenting symptom in 40.5% of female pediatric patients (median age of onset 9 years; Table 6) [8]. GI symptoms, including abdominal pain and diarrhea, are a presenting symptom in 11.4% of female pediatric patients (median age of onset 9.5 years; Table 6) [8], and are associated with significant impairment of quality of life that can be severe enough to cause school absences, decreased participation in physical activities and behavioral problems [8, 109].

**Table 6:** Gastrointestinal symptoms, pain, and health-related quality of life in females with FD versus females in the general population [3, 8, 12, 17, 21, 79, 80, 110, 111, 113, 115, 117–121]

Outcome	Females with FD	Females in the general population
Abdominal pain	45% (*n* = 75/166; Fabry Registry; mean age: 43 years) [17] 60% (*n* = 37/62; mean age: 39.3 years; Netherlands) [110]	24.4% [111] 30% (*n* = 16/53; mean age: 41.7 years; Netherlands) [110]
Diarrhea	39% (reported by adult females; *n* = 63/160; Fabry Registry; mean age at onset: 19.9 years) [17] 11.4% (reported by parents of pediatric patients; *n* = 18/158; Fabry Registry; median age at onset: 9.5 years) [8]	26.7% [111]
Any GI disturbance	11.4% (*n* = 18/158; Fabry Registry; aged < 18 years [median age at onset: 9.5 years) [8] 45% (*n* = 339/754; Fabry Outcome Survey; mean age at onset: 26.8 years) [80]	65% (*n* = 28,804/42,696; ≥ 18 years) [121] 19% (*n* = 10/53; mean age: 41.7 years; Netherlands) [110]
Chronic pain	32.3% (*n* = 51/158; Fabry Registry; aged < 18 years) [8] 32.3% (*n* = 80/248; Fabry Outcome Survey; mean age at onset: 20.7 years) [12] 39.6% (*n* = 25/63; mean age: 45 years; Italy) [118]	5.1% (*n* = 561; aged ≤ 16 years [mean age: 11.9 years]; Spain) [119] 21.7% (≥ 18 years) [120]
Neuropathic pain	40.5% (*n* = 64/158; Fabry Registry; aged < 18 years [median age onset: 9 years]) [8] 43.3% (*n* = 457/1077; Fabry Registry; mean age at onset: 14.2 years) [3] 64% (Fabry Outcome Survey; mean age at onset: 16.9 years) [21]	6.9–24.1% (≥ 18 years) [113]
RAND-36 HRQoL scores	*n* = 202 (mean age: 45 years; US, Canada, UK)[115]	*n* = 8102 (≥ 18 years^a^)^b^^[[115]]^
General health score	45.1	76.5
Role limitations emotional	55.4	85.3
Energy/fatigue	40.4	64.8
Emotional well-being	65.2	79.8
Pain	62.2	77.2
Physical functioning	67.1	82.9
SF-36 questionnaire scores	*N* = 15 (mean age 45 years; Germany) [117]/*N* = 19 (median age 48 years) [79]/*N* = 82 Fabry Registry; age 44– < 54 years) [3]	Germany [117]/*N* = 1421 (≥ 18 years) [79]
General health	41.1/54.1/51.2	66.0/70.6
Vitality	31.7/42.6/46.2	57.6/58.4
Social function	60.0/69.3/70.3	84.2/81.5
Role—emotional	37.8/59.2/67.5	86.7/79.5
Mental health	57.1/65.5/63.8	69.8/73.3
Physical functioning	48.0/62.2/69.9	82.8/87.2
Role—physical	25.0/64.3/56.7	79.2/73.6
Bodily pain	46.5/59.2/63.0	63.9/77.8

FD, Fabry disease; GI, gastrointestinal; HRQoL, health-related quality of life; RAND-36, RAND 36-Item Health Survey questionnaire

^a^Post-menopausal women. ^b^Meta-analysis comprising 19 studies (44–66,616 participants)

GI symptoms and neuropathic pain can be severe and can persist into adulthood. Data from the Fabry Registry suggest that 43% of females experience neuropathic pain, and 45 and 39% experience abdominal pain and diarrhea, respectively [3, 17]. Abdominal pain (45–60 vs. 24–30%) and diarrhea (39–41 vs. 19–27%) have been reported more frequently in females with FD than in the general population (Table 6) [17, 110, 111]. The frequency of abdominal pain in females with FD appears comparable to that in persons with ulcerative colitis (52%; Table 6) [112]. Similarly, neuropathic pain is more frequent in females with FD (41–64%) than in the general population (7–24%; Table 6) [3, 8, 21, 113]. In an international survey including 245 female responders, the intensity of FD-specific pain was described as ‘moderate’ or ‘severe’ by 77.6% (*n* = 190/245), and 81.6% (*n* = 200/245) reporting pain in the hands and feet, with or without accompanying abdominal pain [14]. The pain experienced by females with FD has a disabling effect, commonly interfering with mood, work, sleep, activities, and general enjoyment of life [79, 114].

These FD-associated signs and symptoms have a significant impact on quality of life in females [115], regardless of phenotype [116]. Short Form 36 Health Survey Questionnaire (SF-36) mean scores in females with FD demonstrate significantly worse quality of life when compared with females in the general population (Table 6) [117]. In a US study of 19 females with FD, the largest mean score effects compared with US female population norms were seen in the physical functioning (62.2 versus 87.2, effect size 1.0), general health (54.1 versus 70.6, effect size 0.77), and vitality (42.6 versus 58.4, effect size 0.73) subscales of the SF-36 (Table 6) [79]. In 368 females from the Fabry Registry, scores were significantly lower than age- and sex-matched US norms across all eight SF-36 subscales in persons aged 35–55 years, with fewer subscales revealing significant reductions in younger than older age groups [3]. Female patients aged 14–17 years in the Fabry Registry also had significantly lower scores for the body pain and general health domains of the SF-36 (responses available from 26 patients aged ≥ 14 years) compared with US general population norms for females aged 18–24 years [8].

### Neuropsychiatric impact of FD

The neuropsychiatric impact of FD is frequently under-recognized and, when identified, is often under-addressed and poorly characterized [122]. Reduced health-related quality of life in females with FD may be accompanied by substantial psychological effects, with symptoms of severe depression reported by 22% (*n* = 24/110) of females, of whom 8.3% reported the effects of symptoms of their lives as “extreme” [10]. The probability of suffering from depression is strongly related to the degree to which symptoms of FD (particularly pain, acroparesthesia or anhidrosis) interfere with daily life [10]. More recently, a study of depression in females with FD in the Netherlands reported 38% (*n* = 19/50) females with FD had a documented history of depressive disorder as diagnosed by a psychologist, psychiatrist, or general practitioner [11]. Depression in females with FD may be a symptom of the disease itself or a reaction to living with this chronic condition [123].

High levels of depression have been reported in females with FD; using the Center for Epidemiologic Studies Depression scale (CESD) 39.5% and 30.0% of females with classic and non-classic disease, respectively, were reported to have depressive symptoms (score > 16) [124]. The prevalence of depression in females with FD (22–40%) [10, 124] higher than in women in the US general population (11%) (see Table 7) [125]. In addition, females with FD often experience anxiety, with one study stating that 39% of females (13/33) reported generalized anxiety [79]. Anxiety and depression in females with FD are significantly associated with deficient social adaptive functioning [122]. Furthermore, of 38 adult females with FD, 39.5% reported borderline clinical to clinical range scores on the attention-deficit/hyperactivity scale [126].

**Table 7:** Neuropsychiatric impact in females with FD versus females in the general population [10, 11, 125]

Outcome	Females with FD	Females in the general population
Depression	22%–38% (*n* = 24/110; *n* = 19/50) report symptoms of severe clinical depression	• 10.3% (≥ 18 years) [125]

FD, Fabry disease

Most adult females with FD also report considerable fatigue; according to a case–control survey in 63 heterozygotes and 52 controls, 89% of females with FD and 57% of female controls report fatigue [110]. Although fatigue and decreased energy are often associated with chronic kidney disease or heart problems, FD-related fatigue is reported independent of serious organ involvement [127]. Although the etiology of fatigue in FD is not fully understood, it may be exacerbated by depression and pain, and is significantly associated with impairment of physical health-related quality of life [128].

### Pregnancy

Fabry disease has not been shown to have an impact on reproductive fitness in females. An analysis of self-reported reproductive data in 242 females with FD indicated increased reproductive fitness in females with FD [129]. On average, females with FD had more biological, live-born children than the general population in the United States [129]. While reproductive fitness does not appear to be impaired in females with FD, disease-related manifestations such as GI symptoms, acroparesthesia, proteinuria, headaches, and postpartum depression may worsen during pregnancy. Proteinuria in females with FD may increase the risks of obstetric complications such as pregnancy-associated hypertension, potentially creating a high-risk pregnancy. A study of 41 adult females with FD revealed a statistically significant increased frequency of hypertension observed during pregnancy when compared with the frequency of hypertension in females without FD in the general population (*p* < 0.05) [130]. These data are limited by the small sample size, but suggest that when pregnant, women with FD could experience exacerbation of their FD symptoms and may benefit from early consultation with a maternal–fetal medicine specialist [130].

### Consideration of other factors contributing to phenotypic heterogeneity in females with FD

In addition to phenotype and genetic variant, there are several other biological factors specific to females that may influence disease progression in females with FD and therefore merit investigation. For example, low body weight and post-menopausal status in untreated females with FD have both been associated with the development of skeletal complications including osteoporosis [131]. Consideration of hormone variability throughout life as well as thyroid dysfunction should also be discussed, as both can contribute to the overall risk of disease in females in the general population [132, 133]. The American Thyroid Association reported that one in eight females will experience thyroid dysfunction in their lifetime [134]. The poor management of thyroid function may exacerbate FD-related fatigue and exercise intolerance, and contribute to weight gain and cardiovascular manifestations including tachycardia [134, 135]. Similarly, menopause, which typically affects women between 49 and 52 years of age, increases the risk of cardiovascular disease [136]. Cardiac dysfunction is observed in females with FD at a substantially greater extent than in females in the general population and is the leading cause of mortality for these patients [9, 65]. This highlights the importance of evaluating and managing FD in females beyond their immediate FD-specific organ involvement.

While not specific to females, co-existing risk factors such as diabetes or sarcomeric protein abnormalities should also be considered when evaluating females with FD. Non-FD related medical history may contribute to a more rapid progression of FD, due in part to reduced cardio-kidney reserve.

## Limitations

As previously stated, while the ongoing Fabry Registry and the completed Fabry Outcome Survey, both international, observational, multicenter registries, enrolled females across a comprehensive range of disease severity, there exists the possibility of patient enrollment bias toward the inclusion of more severely affected female patients being followed by registry centers and thus enrolled in registry studies.

## Conclusions

Females with FD experience serious disease manifestations, including cardiovascular symptoms, cerebrovascular complications, kidney impairment, neuropathic and abdominal pain, diarrhea and depression at considerably higher rates than the general population. One of the earliest symptoms in this group is neuropathic pain, with severe pain starting early in life.

In addition to the physical and functional limitations they impose on females, FD-associated symptoms have a significant psychological impact and are associated with substantial impairment of health-related quality of life relative to the general population. They are also associated with reduced life expectancy compared with the general population. The tendency toward comparing females with FD with males, as well as the under-management and under-monitoring of symptomatic females, contributes to the potential for delayed initiation of appropriate management and disease specific therapy. Close monitoring of symptoms and organ involvement in female patients diagnosed with FD is necessary.

Despite these observations, females with FD are often under-represented in clinical trials, and their disease trajectory and symptomatic progression remain poorly documented. Future research should focus on optimizing approaches to comprehensive management of symptoms specifically in females, and improving the identification, monitoring, and care of female patients before overt symptom presentation in order to minimize the lifetime impact of FD. Crucially, there remains an unmet need for clinical studies that focus on and include sufficient numbers of women with FD for clinically and statistically meaningful analyses. Such studies would recruit females only or be prospectively designed and powered for separate analysis of females where recruitment includes both sexes.

## Data Availability

Not applicable.

## Abbreviations

ACR: Albumin–creatinine ratio
AF: Atrial fibrillation
ASIAN-FAME: Asian Fabry Cardiomyopathy High-Risk Screening Study
CESD: Center for Epidemiological Studies Depression
CHD: Coronary heart disease
cMRI: Cardiac magnetic resonance imaging
CHF: Congestive heart failure
CNS: Central nervous system
CV: Cardiovascular
eGFR: Estimated glomerular filtration rate
ESKD: End-stage kidney disease
FD: Fabry disease
GI: Gastrointestinal
GL3: Globotriaosylceramide
HF: Heart failure
HRQoL: Health-related quality of life
IBS: Irritable bowel syndrome
LGE: Late gadolinium enhancement
LVH: Left ventricular hypertrophy
lyso-GL3: Globotriaosylsphingosine
MACE: Major adverse cardiovascular events
MI: Myocardial infarction
MRI: Magnetic resonance imaging
NT-proBNP: N-terminal brain natriuretic propeptide
NVSS: National Vital Statistics System
RAND-36: RAND 36-Item Health Survey questionnaire
KRT: Kidney replacement therapy
SD: Standard deviation
SF-36: Short Form 36 Health Survey Questionnaire
TIA: Transient ischemic attack
Α-Gal A: α-Galactosidase A

## Footnotes

1 Podocytes are terminally differentiated cells of the kidney glomerulus that are essential for the integrity of the kidney filter. Their function is primarily based on their intricate structure, which includes foot processes. Loss of these actin-driven membrane extensions is tightly connected to the presence of protein in the urine, podocyte loss, development of chronic kidney disease and ultimately kidney failure [37].

## Declarations

### Statement of prior presentation

Some data within the manuscript were presented at the 18th Annual WORLD Symposium hybrid (virtual and in-person) congress, February 7–11, 2022.
